# Excision of Nucleopolyhedrovirus Form Transgenic Silkworm Using the CRISPR/Cas9 System

**DOI:** 10.3389/fmicb.2018.00209

**Published:** 2018-02-16

**Authors:** Zhanqi Dong, Feifan Dong, Xinbo Yu, Liang Huang, Yaming Jiang, Zhigang Hu, Peng Chen, Cheng Lu, Minhui Pan

**Affiliations:** ^1^State Key Laboratory of Silkworm Genome Biology, Southwest University, Chongqing, China; ^2^College of Biotechnology, Southwest University, Chongqing, China; ^3^Key Laboratory of Sericultural Biology and Genetic Breeding, Ministry of Agriculture, Southwest University, Chongqing, China

**Keywords:** *Bombyx mori*, BmNPV, CRISPR/Cas9, transgenic, antiviral therapy

## Abstract

The CRISPR/Cas9-mediated genome engineering has been shown to efficiently suppress infection by disrupting genes of the pathogen. We recently constructed transgenic lines expressing CRISPR/Cas9 and the double sgRNA target *Bombyx mori* nucleopolyhedrovirus (BmNPV) *immediate early-1* (*ie-1*) gene in the silkworm, respectively, and obtained four transgenic hybrid lines by G1 generation hybridization: Cas9(-)/sgRNA(-), Cas9(+)/sgRNA(-), Cas9(-)/sgRNA(+), and Cas9(+)/sgRNA(+). We demonstrated that the Cas9(+)/sgRNA(+) transgenic lines effectively edited the target site of the BmNPV genome, and large fragment deletion was observed after BmNPV infection. Further antiviral analysis of the Cas9(+)/sgRNA(+) transgenic lines shows that the median lethal dose (LD50) is 1,000-fold higher than the normal lines after inoculation with occlusion bodies. The analysis of economic characters and off-target efficiency of Cas9(+)/sgRNA(+) transgenic hybrid line showed no significant difference compared with the normal lines. Our findings indicate that CRISPR/Cas9-mediated genome engineering more effectively targets the BmNPV genomes and could be utilized as an insect antiviral treatment.

## Introduction

*Bombyx mori* is a silk insect with high economic, commercial, and cultural value that is currently threatened by various diseases (Jiang and Xia, 2014; Zhang et al., 2014b). *B. mori* nucleopolyhedrovirus (BmNPV) is the most common serious disease that causes huge economic losses in sericulture each year (Zhang et al., 2014a,b). Currently, prevention and control of BmNPV mainly involve conventional sterilization methods, which have been determined to be ineffective in controlling infections (Arakawa et al., 2002; Jiang and Xia, 2014). Improvements in the silkworm mulberry industry have resulted in the generation of transgenic lines with antiviral ability by modern molecular breeding using RNA interference (RNAi) or overexpressing resistance genes (Jiang et al., 2012; Jiang and Xia, 2014; Zhou et al., 2014; Zhang et al., 2014b,c). Although the current antiviral strategy could inhibit baculovirus infection and reduce viral DNA replication, there is still a need to establish effective measures for BmNPV prevention and control (Jiang and Xia, 2014). Genome engineering techniques have been recently applied to genetic improvement studies, including molecular therapy of infectious diseases (Jiang and Xia, 2014).

The recent development of the clustered regularly interspaced short palindromic repeats/CRISPR-associated protein 9 (CRISPR/Cas9) system as a genome editing technology has facilitated modifying the genetic material of pathogens and host cell genes, as well as elucidation of the mechanism underlying infections (Bondy-Denomy et al., 2013; Ledford, 2017; Puschnik et al., 2017). CRISPR/Cas9 has been used in phenotypic whole genome screens, the characterization of gene function, and the identification of potential targets of vaccines against pathogenic bacterial, viruses, parasites, and fungi (Khatodia et al., 2017; Ledford, 2017). Recently, Lin et al. (2015) showed that this system could cleave the intrahepatic HBV genome and facilitate its clearance *in vivo*, as well as excise HIV-1 provirus DNA from the host genome in pre-clinical animal models. In addition, Burkard et al. (2017) generated pigs lacking the CD163 SRCR5 domain that exhibited full resistance to porcine reproductive and respiratory syndrome (Yin C. et al., 2016). The CRISPR/Cas9 system has also been successfully tested in antiviral and gene therapy studies in animal models.

Previously, we constructed a highly efficient CRISPR/Cas9 system to disrupt BmNPV in infected *B. mori* cells (Dong et al., 2016). To determine the gene editing efficiency and antiviral effect of this system in silkworm *in vivo*, here, we further developed Cas9 protein and the double sgRNA targeted BmNPV *immediate early-1* (*ie-1*) gene expression transgenic lines using CRISPR/Cas9 system. The double positive transgenic lines Cas9(+)/sgRNA(+) were obtained by G1 generation hybridization, and further DNA sequencing analysis indicated that the Cas9(+)/sgRNA(+) transgenic lines could efficiently eliminate the BmNPV genome after infection. Antiviral analysis of Cas9(+)/sgRNA(+) transgenic hybrid lines showed a significant increase in survival rate and inhibition of viral gene expression after BmNPV infection. Analysis of economic traits also indicated that the larval weight and cocoon rate of the Cas9(+)/sgRNA(+) lines did not significantly differ from those of the other strains. The findings of the present study indicate that the CRISPR/Cas9 system has the ability to edit the BmNPV genome and suppress virus multiplication in transgenic animals.

## Materials and Methods

### Silkworm Strains and Viruses

The “305” strain of *B. mori* was maintained at our laboratory and used in this study. The silkworm larvae ware reared on fresh mulberry leaves under standard conditions. The silkworm larvae underwent oral inoculation with wild-type (WT) Chongqing strain of BmNPV, and OBs were harvested from the infected hemolymph before the larvae died (Dong et al., 2014). The number of OBs were counted using a Nageotte hemocytometry and stored at 4°C.

### Vector Construction

The Cas9 and sgRNA expression cassettes of the target gene *ie-1* were constructed as described in our previous study (Dong et al., 2016). We selected the two target sites of the BmNPV *ie-1* gene as gene editing sites, which were separated by a 299-bp DNA segment in the BmNPV genome. To avoid off-target effects, the candidate target sites had no more than 12 prm matching sequence in the silkworm genome. After single digestion of pSL1180-IE1-Cas9-Ser-PA by *AscI* restriction endonuclease, the fragment IE1-Cas9-Ser-PA was ligated to the pBac[3 × P3 EGFP afm] vector to obtain the green fluorescent protein transgenic vector pBac[IE1-Cas9-Ser-PA-3 × P3 EGFP afm]. Simultaneously, the two target genes were ligated to the vector pSL1180-fa after single digestion with *BglII*, which were then ligated to a pBac[3 × P3 DsRed afm] vector to generate a red fluorescent protein transgenic vector for pBac[U6-sgRNA-3 × P3 DsRed afm]. All primers used in the study are listed in **Supplementary Table S1**, and all constructed vectors were verified by sequencing.

### Microinjection and Screening

The donor plasmids pBac[IE1-Cas9-Ser-PA-3 × P3 EGFP afm] and pBac[U6-sgRNA-3 × P3 DsRed afm] were mixed with the helper plasmid pHA3PIG at a molar ratio of 1:1 and injected into silkworm eggs in the embryo, with the sites of injection sealed with non-toxic glue, and then placed in a 25°C incubator. The larvae were harvested and fed to the moths, and G1 silkworm eggs were crossed with the parental hybrids of G0. The positive individuals were screened by stereoscopic fluorescence microscopy. Genomic DNA was extracted from the transgenic strains and digested with *HaeIII* overnight at 37°C, then purified and self-ligated with a cyclized fragment. PCR amplification of the ligated product was performed using transposon-specific primers of pBacL and pBacR, and then the amplified product was cloned into a T-cloning vector. The transgenic insertion site was analyzed by sequencing.

### T7 Endonuclease I Assays

Genomic DNA was extracted at difference infection times using a *TransTaq* DNA polymerase high fidelity kit (TransGen Biotech, Beijing, China) according to the manufacturer’s protocol. The target site of the BmNPV genome was amplified with a PCR reagent kit (Takara, Dalian, China) using the indicated primers (**Supplementary Table S1**). Then, the PCR products were reannealed in NEBuffer 2 (NEB, United States) using the following conditions: 95°C for 5 min; 95–85°C at -2°C/s; 85–25°C at -0.1°C/s; hold at 4°C, and then digested with T7 endonuclease I (T7EI) for 30 min at 37°C. After digestion with T7EI, the products were subjected to heat treatment at 60°C for 10 min to inactivate the enzyme. The enzyme-DNA mixtures were subjected to 2% agarose gel electrophoresis, using PCR products without T7EI treatment as negative control. The gels ware stained with ethidium bromide (EB) and imaged using a Bio-Rad Gel Doc gel imaging system (Bio-Rad, United States) by densitometry.

### Sequence Assays

The purified BmNPV genome DNA products were ligated into a pEASY-T5 Zero cloning vector (TransGen Biotech, Beijing, China) and sequenced using M13 primers. All primers used to detect the target gene mutation are presented in **Supplementary Table S1**.

### Off-Target Assays

To study the frequency of off-target editing in the silkworm genome, we assessed the potential occurrence of off-target sites for two sgRNAs through the CRISPR design tools^1^ (Naito et al., 2015). For each sgRNA, we screened the top three sites with off-target frequencies, and then designed the corresponding primers at the target site for PCR amplification. The corresponding PCR product was ligated to the pEASY-T5 Zero cloning vector, then sequenced and aligned with the target gene sequence. All off-target sites primers used in this study are presented in **Supplementary Table S1**.

### Mortality Analyses

The OBs of BmNPV were purified from diseased larvae as previously described (Dong et al., 2014). The transgenic silkworm larvae were reared on fresh mulberry leaves under standard conditions and then fourth instar larvae were inoculated with 1 × 10^3^, 1 × 10^4^, 1 × 10^5^, 2 × 10^5^, 1 × 10^6^, 1 × 10^7^, and 1 × 10^8^ OBs/larva. Per dose were used in each transgenic line at the age of 4 instar larvae, and each dose was performed in triplicate. Infected larvae were reared individually in plates and monitored daily until all of them had either pupated or died.

### Quantitative Real-Time PCR (qPCR) DNA Replication Assay

Genomic DNA extraction followed by quantitative PCR was performed as previously described (Dong et al., 2015, 2017). A DNA standard curve was constructed based on the *C*t value of the serial dilution control sample, which was then used in calculating for GP41 copy number. The q-PCR conditions were as follows: 95°C for 30 s; followed by 40 cycles at 95°C for 5 s, 60°C for 20 s, and using 1 μM of each primer. All experiments were repeated thrice.

### Reverse Transcription-PCR (RT-PCR)

Total RNA was isolated from each transgenic line at 0, 12, 24, 48, 72, 96, and 120 h after inoculation with BmNPV and the corresponding cDNAs were synthesized according to the manufacturer’s protocol (OMEGA, United States). RT-PCR analysis was performed with SYBR Select Master Mix Reagent (Bio-Rad, United States) using primers specific for the *ie-1*, *vp39*, and *poly* genes (**Supplementary Table S1**). The *B. mori* *sw22934* gene was used as control. The RT-PCR conditions were as follows: 95°C for 30 s; followed by 40 cycles at 95°C for 5 s and 60°C for 20 s, using 1 M of each primer. All experiments were performed in triplicate.

### Analysis of Economic Characteristics

Four transgenic hybrid lines, namely, Cas9(-)/sgRNA(-), Cas9(+)/sgRNA(-), Cas9(+)/sgRNA(-), and Cas9(+)/sg RNA(+) were obtained by Cas9 and sgRNA transgenic line hybridization. Each transgenic strain was screened in triplicate, with each replicate consisting of 30 larvae at various developmental stages, from the beginning of the fourth instar until pupation. The average weight of the larvae for each developmental stage was calculated. A total of 30 cocoons of each strain were randomly selected before the moth stage, the total cocoon volume and cocoon size were analyzed, and the cocoon shell rate was calculated. Each transgenic line was determined by the mean of three independent replicates.

### Statistical Analysis

All transgenic lines were screened in triplicate, with each replicates consisting of 30 larvae. All data were expressed as the mean ± SD of three independent experiments. Statistical analyses were performed with the Student’s *t-*test using GraphPad Prism 5. Differences with *P* < 0.01 were considered statistically significant.

## Results

### Construction of a CRISPR/Cas9 System in Silkworm

Genetic engineering of silkworm was performed using the *piggyBac* transposon as vector and fluorescent protein (EGFP or DsRed) as a marker under the control of a promoter that drives expression specifically in the eyes. The principle of transgenes is involves the injection of plasmid DNA harboring a transposon containing a delivery gene and a marker gene into early embryos along with a helper plasmid carrying the P-element transposon. The transgenic silkworm was detected and collected in the next silkworm generation through the expression of the marker gene. Therefore, we selected chose eye-specific promoters 3 × P3 of the silkworm promoter green fluorescent protein (EGFP) and the red fluorescent protein (DsRed) expression plasmid (pBac[3 × P3 EGFP afm] and pBac[3 × P3 DsRed afm]) as the parental vector. To detect the gene editing efficiency of the CRISPR/Cas9 system in silkworm, we constructed the vector pBac[IE1-Cas9-Ser-3 × P3 EGFP afm] and pBac[U6-sgRNA-3 × P3 DsRed afm] to express the Cas9 protein and the sgRNA target sequence, respectively.

**FIGURE 1:**
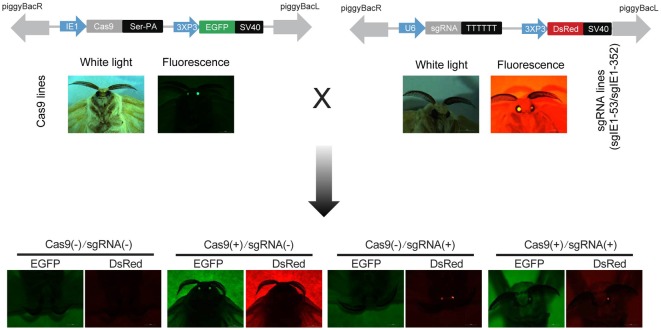
Construction of the CRISPR/Cas9 system of transgenic silkworm. Transgenic vector construction of pBac[IE1-Cas9-Ser-PA-3 × P3 EGFP afm] and pBac[U6-sgRNA-3 × P3 DsRed afm] injection with the helper plasmid. G1 generation of Cas9- and sgRNA-positive strains were detected by fluorescence screening, G2 generation four transgenic hybrid lines, namely, Cas9(–)/sgRNA(–), Cas9(+)/sgRNA(–), Cas9(+)/sgRNA(–), and Cas9(+)/sgRNA(+) were generated by G1 hybridization.

The baculovirus *ie-1* gene is an immediate early gene for viral DNA replication. It is an essential gene for viral DNA replication and plays a transcriptional regulatory role in viral late viral gene expression. Therefore, we selected the *ie-1* gene as the target gene for our gene editing. To effectively influence the transcription and expression of *ie-1* gene, we selected the two target sites at positions 53 and 352 of the *ie-1* gene expression box. Then, we linked target gene of sgIE1-53 and sgIE1-352 to the same vector, hereafter designated as sgRNA (sgIE1-53/sgIE1-352). After transgenic injection, the Cas9- and sgRNA-positive transgenic lines were detected by green and red fluorescence screening, respectively. Four transgenic hybrid lines, namely, Cas9(-)/sgRNA(-), Cas9(+)/sgRNA(-), Cas9(-)/sgRNA(+), and Cas9(+)/sgRNA(+) were obtained by Cas9 and sgRNA transgenic line hybridization (**Figure 1**). The Cas9(-)/sgRNA(-) line did not express the Cas9 protein nor the sgRNA target sequence, Cas9(+)/sgRNA(-) line expressed the Cas9 protein but not sgRNA, the Cas9(-)/sgRNA(+) line expressed the sgRNA target sequence but not Cas9, and the Cas9(+)/sgRNA(+) line expressed both Cas9 protein and sgRNA target sequence.

To ensure that the insertion site of the transgenic lines had no significant effects on individual silkworms, we analyzed the insertion sites of the sgRNA and Cas9 transgenic lines, respectively. These results show that the sgRNA and Cas9 transgenic lines were inserted into nscaf3031 on chromosome 11 and nscaf2874 on chromosome 5, respectively. The Cas9 lines were inserted into the intron of BGIBMGA011786-TA, and sgRNA lines were inserted into the intergenic region of the *B. mori* genome (**Supplementary Table S2**). No significant effects on both sides of the insertion site in the two transgenic lines were observed (**Supplementary Figure S1**).

### CRISPR/Cas9-Mediated BmNPV Site-Specific Point Mutations in Transgenic Silkworm

**FIGURE 2:**
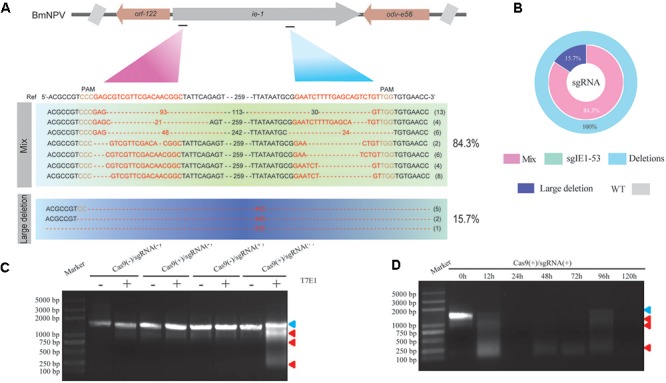
CRISPR/Cas9-mediated BmNPV site-specific point deletion in transgenic silkworm. **(A)** DNA sequencing analysis of the sgIE1-53 and sgIE1-352 target sites in the BmNPV genome. The *ie-1* gene sequence of the WT is shown on top in bold, the target sequence of sgRNA is indicated in red, and the deletion sequence is indicated by dashes. **(B)** The ratios of different types of mutations. **(C)** Determination of editing efficiency of different transgenic lines using T7E1 digestion analysis. The top of the graph represents the transgenic lines, (–) and (+) indicate T7E1 endonuclease. The blue arrow indicates the genome of the target site, and the red arrow shows the mutations induced by T7E1. **(D)** The efficiency of genome editing at different time points after BmNPV infection based on T7E1 digestion analysis.

We selected two target sites in the *ie-1* gene of BmNPV as targets of the sgRNA sequences and ligated sgRNAs targeting these sites into pBac[3 × P3 DsRed afm], followed by determination of the gene editing efficiency of respective sgRNAs in the transgenic hybrid line, Cas9(+)/sgRNA(+). Sequencing showed that both sgRNAs (sgIE1-53 and sgIE1-352) were able to fully edit the *ie-1* gene in the BmNPV genome, with editing efficiency reaching 100% (**Figure 2A**). Simultaneously, we analyzed the sequence mutation rate of 51 sequences, and sgIE1-53 and sgIE1-352 were able to fully edit the target site within the BmNPV genome, in which sgIE1-53 mainly appeared in the absence of 3–93 bp, and sgIE1-352 showed a 10–30 bp deletion at the target site, and with more than 15.7% of large fragments deletions between the two target sites in all sequences (**Figures 2A,B**). Furthermore, only the Cas9(+)/sgRNA(+) lines could edit the BmNPV genome, other transgenic lines did not show the phenomenon of gene fragmentation by T7E1 endonuclease analysis (**Figure 2C**). To further determine the efficiency of gene editing in the Cas9(+)/sgRNA(+) lines, we also analyzed the gene editing efficiency of BmNPV infection at different time points by T7E1 endonuclease analysis. We found that the target gene was extensively edited 12 h after BmNPV infection, and the BmNPV genome was basically fully edited after 24 h post infection (h p.i.) (**Figure 2D**).

To determine whether the two target sites sgIE1-53 and sgIE1-352 had the non-specific gene modification in the silkworm genomes, we further detected the sgIE1-53 and sgIE1-352 non-specific editing sites in transgenic lines after inoculation with BmNPV. T-cloning and sequencing showed no detectable off-target effects in the three top non-specific editing sites of the two target sites (**Table 1**).

**Table 1:** Off-target analysis of the CRISPR/Cas9 system in the transgenic silkworm.

Target site	Off-target site	Matches sequence	Position	Description	Off-target ratio
SgIE1-53	sgIE1-53: GCCGTTGTCGAACGACGCTCGGG	20+PAM	BmNPV:117047-117066		
	OT1-sgIE1-53: TGGGCGAGCTCACGACGCTCAGG	9+PAM	Bmor1:nscaf1108: 594170-594180	BAC clone:007M11	0/23
	OT2-sgIE1-53: GTCAAAGATACACGACGCTCTGG	9+PAM	Bmor1:nscaf1690 2545794-2545804	*Bombyx mori* integrator complex subunit 1	0/19
	OT3-sgIE1-53: GATCAGATACAGCGACGCTCGGG	8+PAM	Bmor1:nscaf1705 751758-751768	Uncharacterized sequence	0/20
sgIE1-352	sgIE1-352: GAATCTTTTGAGCAGTCTGTTGG	20+PAM	BmNPV:117346-117365		
	OT1-sgIE1-352: GCACAATGAGGACAGTCTGTGGG	8+PAM	Bmor1: nscaf1108: 2711777-2711787	Uncharacterized sequence	0/25
	OT2-sgIE1-352: CTAGAGAGGGCTCAGTCTGTAGG	8+PAM	Bmor1:nscaf1516: 205586-205796	Uncharacterized sequence	0/24
	OT3-sgIE1-352: TGACTTGCGAAGCAGTCTGTCGG	10+PAM	Bmor1:nscaf1681: 1090954-1091164	Uncharacterized sequence	0/17

### Improved Resistance in Transgenic Lines Using the CRISPR/Cas9 System

**FIGURE 3:**
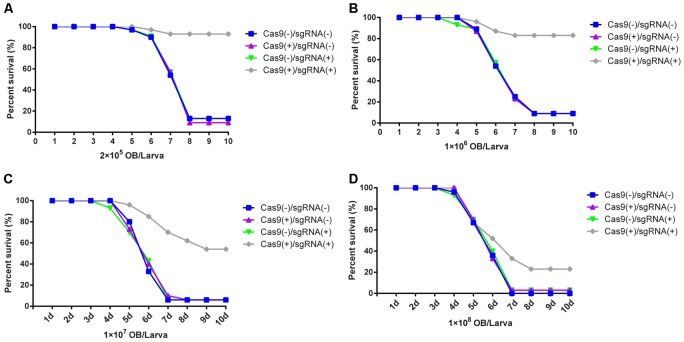
Survival rate analysis of silkworm transgenic lines. Survival rate analysis of transgenic hybrid silkworm lines after inoculation of fourth instar larvae with: **(A)** 2 × 10^5^ OBs/larva, **(B)** 1 × 10^6^ OBs/larva, **(C)** 1 × 10^7^ OBs/larva, and **(D)** 1 × 10^8^ OBs/larva. Each transgenic line was screened in triplicate, each replicate included 30 larvae, and mortality statistical analysis was conducted 10 days after inoculation.

The transgenic hybrid lines Cas9(-)/sgRNA(-), Cas9(+)/ sgRNA(-), Cas9(-)/sgRNA(+), and Cas9(+)/sgRNA(+) were infected by BmNPV by inoculating fourth instar larvae at a density of 2 × 10^5^, 1 × 10^6^, 1 × 10^7^, and 1 × 10^8^ OBs/larva. The results showed that the survival rate of the Cas9(+)/sgRNA(+) lines was still > 95% until the 10th day after inoculation with 2 × 10^5^ OBs/larva, whereas the other three lines exhibited large-scale mortality within 5–8 days after inoculation, and all larvae were dead on the eighth day after OB inoculation (**Figure 3A**). Similarly, after inoculation with 1 × 10^6^ OBs/larva, the survival rate of the Cas9(+)/sgRNA(+) lines was still > 85% on the 10th day, whereas the other hybrid lines died on the seventh day after inoculation with OB (**Figure 3B**). The survival rate of the Cas9(+)/sgRNA(+) lines was still > 54% at 10 days after inoculation with 1 × 10^7^ OBs/larva, whereas almost all of the normal lines had died on the seventh day after OB inoculation (**Figure 3C**). The survival rate of the Cas9(+)/sgRNA(+) lines was only about 33% after inoculation with 1 × 10^8^ OBs/larva, whereas all the control lines had died (**Figure 3D**).

**FIGURE 4:**
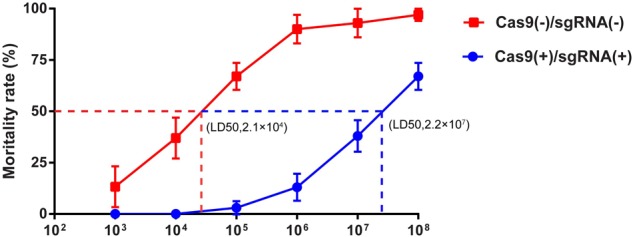
LD50 analysis of transgenic silkworm lines. LD50 analysis of transgenic hybrid lines Cas9(–)/sgRNA(–) and Cas9(+)/sgRNA(+) after inoculation of fourth instar larvae with BmNPV with 1 × 10^3^, 1 × 10^4^, 1 × 10^5^, 1 × 10^6^, 1 × 10^7^, and 1 × 10^8^ OBs/larva, respectively. Each transgenic line was screened in triplicate, with each replicate consisting of 30 larvae. The mortality rate was calculated starting from 4 to 8 days after infection using different OB concentrations.

The LD50 is one of the important markers of resistance in silkworm lines and was thus utilized to determine the resistance of the transgenic lines that we obtained. We tested the LD50 of the transgenic hybrid lines Cas9(-)/sgRNA(-) and Cas9(+)/sgRNA(+) infected with BmNPV by inoculating fourth instar larvae with 1 × 10^3^, 1 × 10^4^, 2 × 10^5^, 1 × 10^6^, 1 × 10^7^, and 1 × 10^8^ OBs/larva using Statistical Product and Service Solutions (SPSS) analysis. The LD50 of the normal lines was 2.1 × 10^4^ OBs/larva, whereas that of the Cas9(+)/sgRNA(+) transgenic lines reached 2.2 × 10^7^ OBs/larva after inoculation of fourth instar larvae with different concentrations of OB, which was more than 1,000-fold higher than the control lines (**Figure 4** and **Supplementary Table S3**). This finding coincided with survival rate analysis, which fully demonstrates that the CRISPR/Cas9 system could effectively improve the antiviral activity of silkworm.

### Evaluation of BmNPV Resistance of Transgenic Lines

**FIGURE 5:**
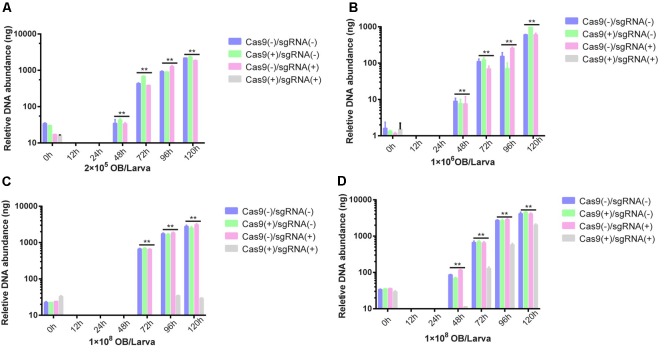
Analysis of BmNPV DNA replication of transgenic lines after OB inoculation. BmNPV DNA replication of four transgenic hybrid lines, namely, Cas9(–)/sgRNA(–), Cas9(+)/sgRNA(–), Cas9(+)/sgRNA(–), and Cas9(+)/sgRNA(+) after BmNPV inoculation of fourth instar larvae using: **(A)** 2 × 10^5^ OBs/larva, **(B)** 1 × 10^6^ OBs/larva, **(C)** 1 × 10^7^ OBs/larva, and **(D)** 1 × 10^8^ OBs/larva. At 0, 12, 24, 48, 72, 96, and 120 h after inoculation, total DNA was isolated from each transgenic line, and Cas9 and sgRNA expression were quantified by Q-PCR. The expression at each time point was determined from the mean of three independent replicates. NS: not significant. ^∗∗^Represents statistically significant differences at *P* < 0.01.

For a more accurate antiviral efficiency analysis of transgenic lines, Cas9(-)/sgRNA(-), Cas9(+)/sgRNA(-), Cas9(+)/sgRNA(-), and Cas9(+)/sgRNA(+) were infected with BmNPV by inoculating fourth instar larvae with 2 × 10^5^ OBs/larva, 1 × 10^6^ OBs/larva, 1 × 10^7^ OBs/larva, and 1 × 10^8^ OBs/larva. At 0, 12, 24, 48, 72, 96, and 120 h after inoculation, total DNA was isolated from each transgenic line and quantified by Q-PCR. The amount of BmNPV DNA was maintained at a low level in the Cas9(+)/sgRNA(+) lines after viral infection, whereas viral DNA abundance continued to rise in the other three lines. Compared to the BmNPV DNA in the other three lines, that in the Cas9(+)/sgRNA(+) lines decreased by 10^4^- to 10^5^-fold (**Figures 5A,B**). Viral DNA abundance slowly increased after inoculation with 1 × 10^7^ OBs/larva, although the difference of the Cas9(+)/sgRNA(+) lines was still 1,000-fold lower than the infected control at different time points (**Figure 5C**). However, viral DNA abundance exhibited a 10-fold increase compared to the BmNPV-infected control after inoculation with 1 × 10^7^ OBs/larva (**Figure 5D**).

**FIGURE 6:**
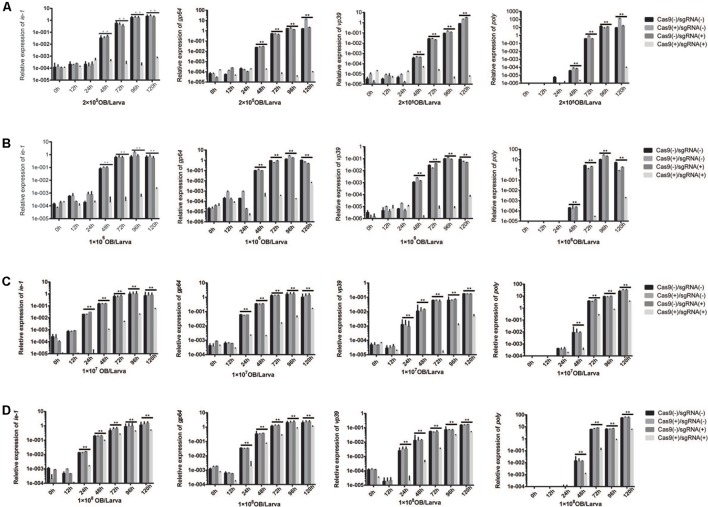
Relative expression levels of BmNPV in transgenic lines at different developmental phases. The relative expression levels of four transgenic hybrid lines, namely, Cas9(–)/sgRNA(–), Cas9(+)/sgRNA(–), Cas9(+)/sgRNA(–), and Cas9(+)/sgRNA(+) after BmNPV inoculation of fourth instar larvae with: **(A)** 2 × 10^5^ OBs/larva, **(B)** 1 × 10^6^ OBs/larva, **(C)** 1 × 10^7^ OBs/larva, and **(D)** 1 × 10^8^ OBs/larva. At 0, 12, 24, 48, 72, 96, and 120 h after inoculation, total RNA from each transgenic line was isolated and BmNPV *ie-1*, *gp64*, *vp39*, and *poly* gene expression levels were quantified by Q-PCR. The expression level of each gene at each time point was determined from the mean of three independent replicates. NS: not significant. ^∗∗^Represents statistically significant differences at the level of *P* < 0.01.

Simultaneously, total RNA was extracted from different transgenic lines, and the expression levels of BmNPV *ie-1*, early gene *gp64*, late gene *vp39*, and very late gene *poly* were analyzed by RT-PCR. The expression of the BmNPV *ie-1*, *gp64*, *vp39*, and *poly* genes were maintained at a very low level in the Cas9(+)/sgRNA(+) lines after inoculation with 2 × 10^5^ OBs/larva and 1 × 10^6^ OBs/larva, whereas these increased in the other three lines (**Figures 6A,B**). The gene expression levels in the Cas9(+)/sgRNA(+) lines was 10^4^- to 10^6^-fold lower than that of the other three lines (**Figures 6A,B**). The transgenic Cas9(+)/sgRNA(+) lines showed the same results after inoculation of fourth instar larvae with 1 × 10^7^ and 1 × 10^8^ OBs/larva, respectively (**Figures 6C,D**). Its expression levels were only about 10- to 1,000-fold lower than the controls after OB inoculation. However, compared to the antiviral effects of the Cas9(-)/sgRNA(-) lines, the differences in expression levels were statistically significant.

### Economic Characteristics of Transgenic Silkworm Lines

**FIGURE 7:**
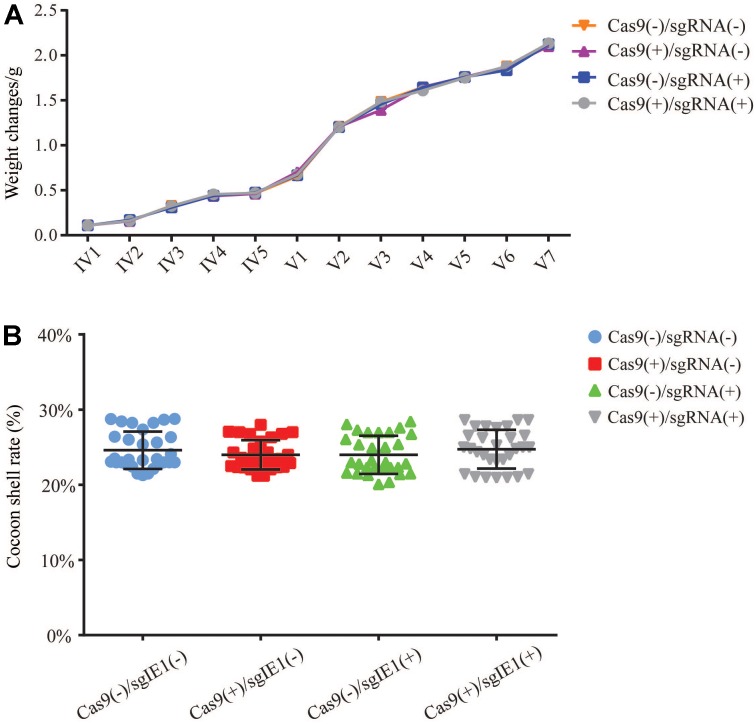
Economic characteristics of transgenic lines. **(A)** Weight changes in the transgenic hybrid lines Cas9(–)/sgRNA(–), Cas9(+)/sgRNA(–), Cas9(+)/sgRNA(–), and Cas9(+)/sgRNA(+) at the fourth and fifth instar larval stages. IV Represents 4 instar larvae, and V Represents 5 instar larvae. **(B)** Analysis of the cocoon shell rate of the transgenic hybrid lines. Each value represents the average of 30 repeated measurements.

To determine the impact of the transgenic Cas9(-)/sgRNA(-), Cas9(+)/sgRNA(-), Cas9(+)/sgRNA(-), and Cas9sg(+)/ RNA(+) lines on growth status and economic characteristics, we analyzed changes in weight of fourth instar larvae of the transgenic lines. Each transgenic lines statistics of 30 larval, repeated three times, and continued statistics to the pupal stage, calculated the average weight. The average weight of the fourth instar larvae was about 0.1 g, the fifth instar larvae weighed 0.5–0.7 g, and before the pupal stage was about 2.0–2.2 g; no significant differences in average weight were observed among the four transgenic lines (**Figure 7A**). The cocoon shell rate of the four transgenic lines ranged from 22 to 26%, with no significant differences among the four lines (**Figure 7B**).

## Discussion

The CRISPR/Cas9 is a new and effective genetic editing tool that has been widely used in gene knockout and knockin studies, genome-wide screening, construction of animal models, gene therapy, antimicrobial and antiviral applications, agricultural production, biological control, and various other areas (Lijuan et al., 2015; Yin H. et al., 2016; Khatodia et al., 2017; Ledford, 2017). CRISPR/Cas9 has also been used in determining the function of virulence factors in hepatitis B virus and *Toxosplasma gondii*, HIV host regulatory proteins, bacterial factors, and the engineering of vaccines for infectious diseases (Zheng et al., 2015; Han and Li, 2016; Seeger and Sohn, 2016; Ledford, 2017; Park et al., 2017). We previously reported that the system can effectively edit the baculovirus genome to inhibit its proliferation in insect cells (Dong et al., 2016). In the present study, we constructed transgenic lines that expressed Cas9 protein and the sgRNA targeting the baculovirus *ie-1* gene, and determined that the transgenic hybrid lines Cas9(+)/sgRNA(+) could inhibit BmNPV proliferation without affecting its economic characteristics. The successful establishment of the antiviral transgenic line Cas9(+)/sgRNA(+) using the CRISPR/Cas9 system provides a novel approach in the biological control of harmful insects.

As early as 2004, RNAi of BmNPV key genes and the overexpression of antiviral proteins have been used to generate silkworm transgenic antiviral lines as a potential therapeutic approach (Isobe et al., 2004). However, it is generally difficult to substantially increase the antiviral effect, thereby hindering its application to sericulture production and performing further in-depth studies (Jiang et al., 2012). Dong et al. (2016) developed the first CRISPR/Cas9 transgenic cell line that could completely inhibit viral gene transcription, protein expression, and BmNPV DNA replication in insect cells. In the present study, we show by BmNPV DNA replication and BmNPV gene expression analysis that the Cas9(+)/sgRNA(+) transgenic hybrids line are capable of inhibiting BmNPV DNA replication. LD50 analysis further confirmed that the Cas9(+)/sgRNA(+) lines can efficiently inhibit BmNPV proliferation to a density of 2.2 × 10^7^ OBs/larva, compared to the control of Cas9(-)/sgRNA(-) lines, which showed the antiviral ability increased by 1,000-fold (**Figure 4**). To our knowledge, this Cas9(+)/sgRNA(+) transgenic line shows the highest antiviral activity, compared to those of previous reports, we find that our articles are very different from the previous report mainly as follows: (1) Our constructed Cas9 and sgRNA transgenic lines were more conducive to sericulture; hybrid progenies with high editing efficiencies could significantly reduce the disruptive effects of BmNPV infection on the growth and development silkworm lines (**Figure 1**); (2) The target site that we selected is closer to the transcriptional start site of the *ie-1* gene expression cassette, which in turn more effectively affects the function of the *ie-1* gene in BmNPV (**Figures 1**, **2**); (3) According to our sequencing results, the final editing efficiency of our CRISPR/Cas9 gene editing system can reach up to 100%, which is not shown in the previous report. A 100% editing efficiency is thus more effective in inhibiting BmNPV DNA replication (**Figure 2**); (4) To the best of our knowledge, our Cas9(+)/sgRNA(+) transgenic line shows the highest antiviral activity compared to those of previous reports, wherein antiviral ability only increased by 10-fold, and may be attributable to the higher target site editing efficiency of our design (**Figures 3**, **4**); (5) In our results, we further show that the individual transgenic lines did not influence silkworm development after gene editing fourth and fifth instar larvae, nor silk yield (**Figure 7**). In summary, our research results are more reasonable and show more extensive inhibition of BmNPV DNA replication. We also more systematically analyzed the inhibition of BmNPV DNA replication (**Figures 5**, **6**) (Chen et al., 2017). The significant antiviral effects of the CRISPR/Cas9 system for the molecular breeding of silkworm may thus be applied to sericulture.

The transgenic antiviral lines used in agricultural production must consider the effects of gene editing on host individuals as well as biosafety (Jiang et al., 2012; Jiang and Xia, 2014). To avoid the effect of off-target effects on the host, we considered the specificity of sgIE1-53 and sgIE1-352 in target gene editing, excluding the off-target sites of our designed sgRNA. To validate the sgRNA specificity of our design, we analyzed the editing efficiency of suspected off-target sites by T-cloning. Sequencing showed that six of the suspected sites do not confer off-target effects (**Table 1**). Transgenic insertion sites may also influence the expression of the host gene. Our analysis of the transgenic sgRNA and Cas9 lines did not detect any gene expression near the insertion site, thereby suggesting that the insertion site does not affect the host (**Supplementary Figure S1**). The silkworm industry is concerned about the effects of transgenic antiviral lines on growth and development as well as cocoon shell rate. The individual transgenic lines did not influence silkworm development after the gene editing of the fourth and fifth instar larvae, nor silk yield. The findings of the present study indicate that the silkworm antiviral line can effectively inhibit BmNPV replication without impacting its host, thereby suggesting that this approach may be applied to the future sericulture production.

## Conclusion

We developed transgenic antiviral lines that show high BmNPV resistance, which may improve current silkworm production and be utilized as a treatment regimen for infections. In the future, we will further optimize the CRISPR/Cas9 system by changing the gene editing target and increasing the editing efficiency in silkworms. The success of this study has also shed light on the prevention and control of insect pathogens and the use of the system to effectively reduce the spread of harmful insects.

## Author Contributions

ZD, FD, XY, and LH performed vector cloning, sequencing, cell culturing, and PCR. ZD, FD, and XY conducted the transgenic injections. YJ and ZH performed the mortality analyses and the DNA replication assay. ZD, MP, and CL conceived the experimental design and participated in data analysis. ZD, MP, PC, and CL prepared the manuscript. The final manuscript was reviewed and approved by all authors.

## Conflict of Interest Statement

The authors declare that the research was conducted in the absence of any commercial or financial relationships that could be construed as a potential conflict of interest.

## Footnotes

1 http://crispr.dbcls.jp/

## Supplementary Material

FIGURE S1 Analysis of transcription levels of genes within the insertion site. **(A)** Transcription levels of BGIBMGA011754 and BGIBMGA0011755 in transgenic sgIE1 and control silkworm. **(B)** Transcription levels of BGIBMGA011786, BGIBMGA011689, and BGIBMGA0011690 in transgenic Cas9 and control silkworm.

TABLE S1 Sequences of primers used in this study.

TABLE S2 Analysis of transgenic silkworm insertion sites.

TABLE S3 LD50 analysis of transgenic silkworm lines.
