# Supplementary figures and images for: Excision of Nucleopolyhedrovirus Form Transgenic Silkworm Using the CRISPR/Cas9 System
Source: Front Microbiol. 2018 Feb 16;9:209. doi: 10.3389/fmicb.2018.00209 (PMC5820291; doi:10.3389/fmicb.2018.00209)

A

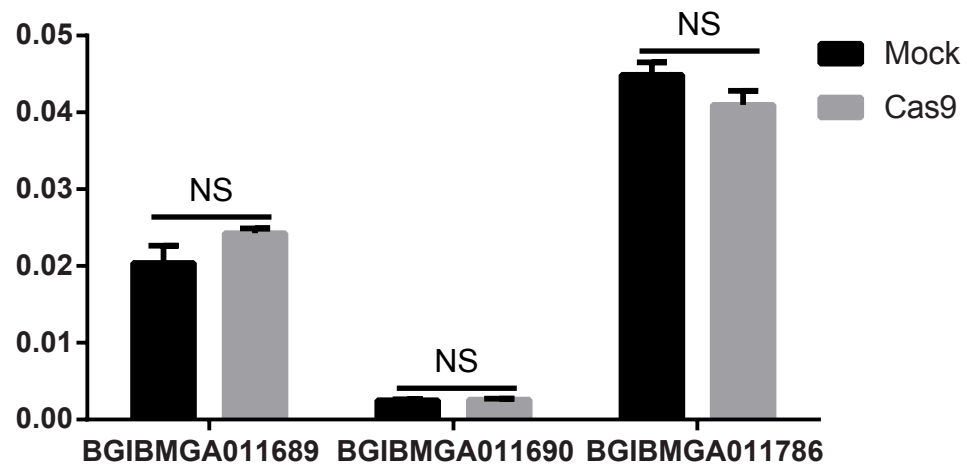

B

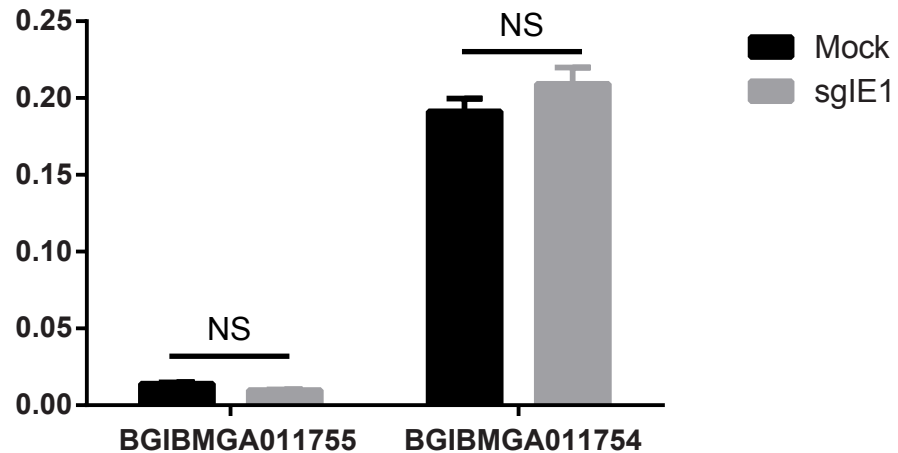

Supplement: FIGURE S1 — Analysis of transcription levels of genes within the insertion site. (A) Transcription levels of BGIBMGA011754 and BGIBMGA0011755 in transgenic sgIE1 and control silkworm. (B) Transcription levels of BGIBMGA011786, BGIBMGA011689, and BGIBMGA0011690 in transgenic Cas9 and control silkworm. [file Image_1.pdf]
