# Supplementary material for: Excision of Nucleopolyhedrovirus Form Transgenic Silkworm Using the CRISPR/Cas9 System
Source: Front Microbiol. 2018 Feb 16;9:209. doi: 10.3389/fmicb.2018.00209 (PMC5820291; doi:10.3389/fmicb.2018.00209)
Supplement: TABLE S1 — Sequences of primers used in this study. [file Table_1.DOCX]

**Supplementary Table 1. Sequences of primers used in this study.**

| Primer name | Primer sequence^*^ |
| --- | --- |
| sgRNA primers | |
| U6-Bgl II/F | 5' CCGCTCGAGAGGTTATGTAGTACACATT 3' |
| U6-Bgl II/R | 5' TTACCGGTAAAAAAAGCACCGACTCG 3' |
| sgIE1-53/F | 5' AAGTGCCGTTGTCGAACGACGCTC 3' |
| sgIE1-53/R | 5' AAACGAGCGTCGTTCGACAACGGC 3' |
| sgIE1-352/F | 5' AAGTGAATCTTTTGAGCAGTCTGT 3' |
| sgIE1-352/R | 5' AAACACAGACTGCTCAAAAGATTC 3' |
| CRISPR/Cas9 system primers | |
| IE-1^prm^-EcoR I/F | 5' AAGCTTTTGCAGTTCGGGAC 3' |
| IE-1^prm^-Cla I/R | 5' CCATCGATTAGATCCCTAGTCG 3' |
| Cas9-Cla I/F | 5' CCATCGATATGGACTATAAGGACCAC 3' |
| Cas9-Xba I/R | 5' GCTCTAGAGTCGCCTCCCAGCTGA 3' |
| SV40-NcoI/F | 5' CATGCCATGGGACTCTAGATCATAATC 3' |
| SV40-EcoR I/R | 5' CGGAATTCTACATTGATGAGTTTGGACA 3' |
| RT-PCR primers | |
| RT-IE1/F | 5' CGAGACGGCTGCACAAAA 3' |
| RT-IE1/R | 5' TGCCCAAAAGAAACCCACA 3' |
| RT-VP39/F | 5' AGACACCACAAACCCGAACAC 3' |
| RT-VP39/R | 5' TTGATCGCCAACACCACCT 3' |
| RT-Poly/F | 5' GCAGTGTGAAACCCGATACCAT 3' |
| RT-Poly/R | 5' CCACCTAAGAGCGTGTTGAGC 3' |
| RT-GP64/F | 5' CACCATCGTGGAGACGGACTAC 3' |
| RT-GP64/R | 5' ACCTCGCACTGCTGCCTGA 3' |
| RT-11754/F | 5' CTTACAGTGCTCGTGGTT 3' |
| RT-11754/R | 5' GTTAGTAGTGCCTCGTTCA 3' |
| RT-11755/F | 5' ACAGTATCCGCAATCATCC 3' |
| RT-11755/R | 5' CGAGGTCCAGTAGTAAGAAG 3' |
| RT-11786/F | 5' CATTGTTGACCAAGTTCTGA 3' |
| RT-11786/R | 5' GTGGAGCCGTAACTATCAT 3' |
| RT-11689/F | 5' TTGCTGATGTGCTCACTT 3' |
| RT-11689/R | 5' ATTGCTCTGGCTTCTTCTAT 3' |
| RT-11690/F | 5' GAAGCCTATGTAGCAGAGTAT 3' |
| RT-11690/R | 5' GGAGGTGGTGGTGGTATA 3' |
| GP41 primers | |
| GP41/F | 5' CCTATTCTGTGCTGGTGGTGG 3' |
| GP41/R | 5' ATGTTGATGTGCGGAAAGC 3' |
| Detection primers | |
| IE1-F1 | 5' ATGACGCAAATTAATTTTAAC 3' |
| IE1-R640 | 5' GGTCGGAGAACCTGTTGGAA 3' |
| pBacL F | 5' ATCAGTGACACTTACCGCATTGACA 3' |
| pBacL R | 5' TGACGAGCTTGTTGGTGAGGATTCT 3' |
| pBacR F | 5' TACGCATGATTATCTTTAACGTA 3' |
| pBacR R | 5' GTACTGTCATCTGATGTACCAGG 3' |
| Off-target primers | |
| OT1-sgIE1-53F | 5' GATCGTCTGCCTACAAGGG 3' |
| OT1-sgIE1-53R | 5' TCCTGTGTGGTATTAAAGACTG 3' |
| OT2-sgIE1-53F | 5' GTCAATCACCAGCTCTTCAACTA 3' |
| OT2-sgIE1-53R | 5' ACCTAGTGCAGTGTCTGTGAG 3' |
| OT3-sgIE1-53F | 5' CTCTATTTTATGACAATACAAGTTG 3' |
| OT3-sgIE1-53R | 5' CAGGTACTCAAGGATTACTTATT 3' |
| OT1-sgIE1-352F | 5' AGAACCGCTGTCAGTAGCTCGT 3' |
| OT1-sgIE1-352R | 5' GTACGGTACGGTAGGATCACG 3' |
| OT2-sgIE1-352F | 5' GAAATTATTTACTTCTTGAATGAAA 3' |
| OT2-sgIE1-352R | 5' GCATACGGGCACCTTTTTG 3' |
| OT3-sgIE1-352F | 5' TGACTGTTTCTTTAAAAACCTAA 3' |
| OT3-sgIE1-352R | 5' ATTTATTGTAATAAATAGCAATAAG 3' |

^*^ (The restriction enzyme sites are marked in red).
