# Supplementary material for: Excision of Nucleopolyhedrovirus Form Transgenic Silkworm Using the CRISPR/Cas9 System
Source: Front Microbiol. 2018 Feb 16;9:209. doi: 10.3389/fmicb.2018.00209 (PMC5820291; doi:10.3389/fmicb.2018.00209)
Supplement: TABLE S2 — Analysis of transgenic silkworm insertion sites. [file Table_2.DOCX]

| **Transgenic lines** | **Insertion sites** | **Sequences** | **Insertion Gene** |  |
| --- | --- | --- | --- | --- |
| pBac[IE1-Cas9-Ser-3×P3 EGFP afm] | Chromosome 11 nscaf3031 | CAAAGTCTCGTTTATTCTGT**TTAApiggyBacTTAA**GACTTCCCCACTTTCGAATG | Introns | |
| pBac[U6-sgRNA-3×P3 DsRed afm] | Chromosome 5 nscaf2874 | CTTATGTAAAAAACGTTTTC**TTAApiggyBacTTAA**AGTATTTCGTAATAAATAGA | NO |  |

**Supplementary Table 2. Analysis of transgenic silkworm insertion sites.**
