# Supplementary material for: Excision of Nucleopolyhedrovirus Form Transgenic Silkworm Using the CRISPR/Cas9 System
Source: Front Microbiol. 2018 Feb 16;9:209. doi: 10.3389/fmicb.2018.00209 (PMC5820291; doi:10.3389/fmicb.2018.00209)
Supplement: TABLE S3 — LD50 analysis of transgenic silkworm lines. [file Table_3.docx]

| **Transgenic Lines** | **LD50** | **95% Confidence Interval** |
| --- | --- | --- |
| Cas9(-)/sgRNA(-) | 2.1×10^4^ | 1.3×10^4^～3.3×10^4^ |
| Cas9(+/sgRNA(+) | 2.2×10^7^ | 1.03×10^7^～6.0×10^7^ |

**Supplementary Table 3 LD50 analysis of transgenic silkworm lines.**
